# Histologic grade and STAS as key predictors of distant recurrence in resected early-stage lung adenocarcinoma: a single-center study

**DOI:** 10.3389/fonc.2025.1626863

**Published:** 2025-09-15

**Authors:** Alessandro Bonis, Giulia Pagliarini, Giovanni Maria Comacchio, Marco Mammana, Federica Pezzuto, Vincenzo Verzeletti, Enrica Pellizzer, Alessandro Berni, Stefano Silvestrin, Giorgio Cannone, Eleonora Faccioli, Alessandro Rebusso, Marco Schiavon, Samuele Nicotra, Andrea Dell’Amore, Fiorella Calabrese, Federico Rea

**Affiliations:** ^1^ Thoracic Surgery Unit, Department of Cardiac, Thoracic, Vascular Sciences and Public Health (DSCTV), University of Padova, Padova, Italy; ^2^ Pathology Unit, Department of Cardiac, Thoracic, Vascular Sciences and Public Health (DSCTV), University of Padova, Padova, Italy

**Keywords:** distant recurrence, relapse, lung cancer, adenocarcinoma, pathological predictors

## Abstract

**Introduction:**

Early-stage lung adenocarcinoma (ADC) is curable by surgical resection in most cases. However, unexpectedly, some patients experience distant disease relapse. Emerging evidence suggests that microscopic tumor characteristics may increase the risk of tumor relapse. Consequently, we aimed to test different microscopic variables to assess their association with distant recurrence (DR).

**Materials and methods:**

We retrieved all cases of radically treated stage I-IIA ADCs from 2016 to 2020. Clinical and pathological variables were assessed for their association with DR using univariable and multivariable logistic regression. An EGFR-adjusted model was also provided.

**Results:**

A total of 259 patients were treated (214 lobectomies and 45 segmentectomies). After resection, 54 patients relapsed, 28 of whom had distant recurrences (DR). Spread through air spaces (STAS) was detected in 48% of samples, while vascular invasion (VI) was present in 53%, occurring 17% more frequently in those with DR. Tumor size was larger in patients with recurrence, with the largest tumors observed in those with local recurrence (25.5 mm in local vs. 23.5 mm in DR; p=0.028). Dedifferentiated (G3) ADCs were more prevalent in DR cases, accounting for 48% of samples. In univariate regression, surgical margins, LVI, necrosis, G3 primary tumors, and STAS were significant factors. In multivariate analysis, STAS showed a trend towards significance (p=0.07) while G3 remained decisive (p<0.01). The EGFR-adjusted model for DR yielded slightly better results (p=0.05 and p<0.01 respectively).

**Conclusions:**

Dedifferentiation and partially STAS are key pathological predictor of distant recurrence in resected stage I-IIA ADCs. The contribution of LVI and tumor necrosis in DR needs to be further clarified. Tumor aggressiveness goes beyond the simple size measurement, claiming for a reassessment of risk models for recurrence after surgery.

## 1 Introduction

Lung cancer remains one of the leading causes of cancer-related mortality worldwide, due to its aggressive nature and late-stage diagnosis in many patients (1). Despite this, the implementation of lung cancer screening programs, advances in neoadjuvant therapies, and the identification of novel targetable driver mutations offer promising avenues for improving the management of non-small cell lung cancer (NSCLC) (2, 3). However, cancer relapse remains a major clinical challenge, as even in localized cases, recurrence occurs in 20-50% of patients despite adequate and complete resection. Moreover, recurrence within one year is one of the most significant negative prognostic factors for survival, highlighting the need for better risk stratification and adjuvant treatment strategies (4). Evidence suggests that other factors beyond the tumor size and the achievement of an R0 resection must also be considered as important predictors of recurrence (5). Features such as histologic grade, vascular and lymphatic invasion (LVI), spread through air spaces (STAS), and tumor microenvironment characteristics have been increasingly recognized for their role in tumor aggressiveness and metastatic potential (6). This study aimed to evaluate the relationship between microscopic tumor features and distant recurrence after curative-intent resection of early-stage lung adenocarcinoma. The goal was to identify histopathological factors beyond tumor size and resection status that contribute to distant recurrence risk, thereby enhancing risk stratification and improving prognostic assessment in stage I-IIA ADCs.

## 2 Materials and methods

This monocentric retrospective study was conducted at the Thoracic Surgery Unit, University of Padova, Italy. Data were analyzed from a consecutive cohort of patients who underwent an upfront resection for ADC between January 1, 2016, and December 31, 2020. The study complied with the principles of the Declaration of Helsinki, and all patients provided written informed consent for participation in the department’s research activities before surgery. The study design received approval from the local Ethics Committee (PD n°0038657, 31/05/2024).

### 2.1 Inclusion criteria

The study included patients over 18 years of age with primary ADC (p-stage I-IIA, 8th AJCC TNM classification) who underwent lobectomy or segmentectomy with radical hilar and mediastinal lymphadenectomy. A preoperative diagnosis of ADC was confirmed by CT-guided or bronchoscopic biopsy, or through postoperative pathological assessment.

Patients with benign or secondary pulmonary lesions were excluded. To enhance sample comparability in line with previous studies, non-anatomical resections (e.g., wedge resections) were also excluded.

### 2.2 Data retrieval

Data were collected from the perioperative setting, including registry information such as age at surgery, gender, body mass index (BMI), comorbidities, and smoking history. Preoperative pulmonary function tests (Forced Vital Capacity - FVC, Forced Expiratory Volume in one second - FEV1, and alveolar carbon monoxide diffusion limit - DLCO/VA) were performed to evaluate operative tolerance and predicted postoperative risk. Additional data included operative time, type of resection, and intraoperative details. Postoperative follow-up data were gathered from outpatient visits, radiological assessment, and clinical evaluations. In cases of missing follow-up information, data was obtained via phone contact or censored at the last available visit.

### 2.3 Pathological examination

Pathological data were derived from resected specimens and included growth pattern, histologic grade (G1 to G3), necrosis (<10%, 10-30%, >30%), and tumor-infiltrating lymphocytes (TILs, <10%, 10-30%, >30%). The presence of Spread Through Air Spaces (STAS), lymphovascular invasion (LVI), and pleural invasion were also assessed. Surgical margins were assessed by pathologists based on the distance between the tumor surface and the nearest structure (parenchymal suture or closed bronchus, artery, or vein), with the mechanically closed 2 mm space from stapler sutures excluded. Margins were considered adequate if they measured greater than 20 mm or, if smaller than 20 mm, exceeded the tumor diameter. PD-L1 expression was quantified using the tumor proportion score (TPS, %), and pathological TNM staging was assigned according to the 8^th^ edition of the AJCC TNM classification.

### 2.4 Surveillance

Follow-up was conducted through regular contrast-enhanced CT scans at 3, 6, or 12 months, in accordance with established guidelines (7). For patients with impaired renal function, non-contrast chest CT scans were approved on a case-by-case basis. After three years, surveillance was performed annually with chest and upper abdominal CT scans. Cerebral MRI was requested only in case of suspected encephalic recurrence. Any suspicion of local or distant recurrence was promptly reviewed by the oncological tumor board.

Locoregional recurrence was defined as any recurrence involving the parenchymal, vascular, or bronchial suture line, ipsilateral hilar and/or mediastinal lymph nodes, or the same lung. Distant recurrence included recurrence in ipsilateral or contralateral serous membranes (pleura, pericardium), the contralateral lung, contralateral hilar and/or mediastinal lymph nodes, the interscalene space, or other extra-thoracic organs. Simultaneous locoregional and distant recurrence was considered as distant recurrence. Upon confirmation, treatment plans and follow-up schedules were determined. Disease-Free Survival (DFS) was defined as the time from surgery to the confirmation of any radiological recurrence (locoregional or distant), as validated by the oncological tumor board.

### 2.5 Statistical analysis

Continuous variables were reported as median and interquartile range (IQR), while categorical variables were presented as absolute numbers and percentages. Group comparisons were performed using Fisher’s exact test, Pearson’s chi-squared test, or Wilcoxon’s test, as appropriate. Univariable logistic regression was used to analyze associations between clinical and pathological variables and distant recurrence. Significant variables were subsequently included in a multivariable logistic regression model. Stepwise elimination based on the Akaike Information Criterion was used to identify the most parsimonious model. Missing data were addressed using multiple imputation. Considering that the EGFR molecular status is a possible confounder due to its relevance in the clincal practice, we intentionally analyzed data introducing the EGFR status into a EGFR-adjusted multivariate model.

All statistical analyses were performed using R software (R Foundation for Statistical Computing, Vienna, Austria).

## 3 Results

A total of 259 patients were included in the study, with a median age of 70 years (IQR 63-75). Of these, 131 (51%) were female, and 191 (74%) had a history of smoking. Hypertension was the most common comorbidity, affecting 148 patients (57%), followed by diabetes (31 patients, 12%) and chronic obstructive pulmonary disease (COPD) (33 patients, 13%). All patients had sufficient respiratory function to undergo lobectomy. Surgical procedures included 214 lobectomies, with a slight predominance of right upper lobectomies (n=92, 36%), and 45 segmentectomies, primarily involving the left upper lobe (14 upper trisegmentectomies, 5.4%) (**Table 1**).

**Table 1:** Population and perioperative data.

Demographic and perioperative data	
Characteristic	N= 259
Age in years	70 (63, 75)
Gender (Females)	131 (51%)
BMI (kg/m^2)	25.7 (23.1, 28.4)
Diabetes	31 (12%)
COPD	33 (13%)
Hypertension	148 (57%)
Smoking history (active or former)	191 (74%)
FVC %	99 (88, 112)
FEV1 %	100 (83, 113)
DLCO/VA %	77 (66, 90)
Surgical time (mins)	125 (95, 115)
Resection type	
RUL	92 (36%)
ML	13 (5.0 %)
RLL	40 (15 %)
LUL	52 (20 %)
LLL	17 (6.6 %)
Right Upper Trisegmentectomy	14 (5.4 %)
Lingulectomy	6 (2.3 %)
Apical segment of the base (S6)	8 (3.1 %)
Basal Segments	4 (1.5 %)
Right S1 or S1+S2	7 (2.7 %)
Right S3	2 (0. 8 %)
S9-S10	4 (1.5 %)
Converted procedures	4 (1.6%)

N (%) or median (IQR). BMI: Body Mass Index, COPD: Chronic Obstructive Pulmonary Disease, FEV1%: Forced Expiratory Volume in 1 s (percentage), FVC%: Forced Vital Capacity (percentage), DLCO/VA%: blood transfer coefficient for the diffusion of CO (percentage). RUL: right upper lobe, ML: middle lobe, RLL: right lower lobe, LUL: left upper lobe, LLL: left lower lobe. S refers to the Segment.

Pathological findings showed a median tumor diameter of 21 mm (IQR 15-30), with the most common pathological stages being IB (n=121, 47%) and IA2 (n=64, 25%). STAS was identified in 124 cases (48%), and the majority of tumors were moderately differentiated (G2, n=189, 74%). Lymphovascular invasion (LVI) was observed in 57 cases (22%), while microscopic pleural involvement as observed in 53% of patients. Only 13 cases (5.5%) exhibited cancer beyond the mesotelial pleural layer (PL2). The median surgical margin was 25 mm (IQR 15-40).

Details of all pathological data are summarized in **Table 2**. The distribution of perioperative and pathological data did not show any significant difference between patients who experienced recurrence and those who remained disease-free during the surveillance (**Supplementary Materials** – **Supplementary Table S1** and **Supplementary Table S2**).

**Table 2:** Pathological data retrieved by the final pathological report.

Pathological variables	
Characteristic	N= 259
T size (mm)	21 (15, 30)
N° harvested lymphonodes (n)	8 (6, 12)
N1 lymphonodes (n)	4 (3, 6)
N2 lymphonodes (n)	4 (3, 6)
STAGE	
IA1	46 (18%)
IA2	64 (25%)
IA3	15 (5.8%)
IB	121 (47%)
IIA	13 (5.0%)
STAS	124 (48%)
Resection margin (mm)	25 (15, 40)
Grading	
G1	6 (2.3%)
G2	189 (74%)
G3	60 (23 %)
Tumor necrosis	35 (14 %)
TILs >10%	153 (60%)
Tumor fibrosis	124 (53 %)
LVI	57 (22 %)
Pleural Invasion (PL)	
PL0	97 (41%)
PL1	126 (53%)
PL2	13 (5.5 %)
EGFR mutation	47 (19%)
Overall surveillance time (months)	54 (40-73)
Surveillance time (in patients with DR)	20 (9-45)

N (%) or median (IQR). STAS: Spread Through Air Spaces; TILs: Tumor Infiltrating Lymphocytes;LVI: lympho-vascular invasion;PL: Pleural invasion (0 = Neoplasm-free pleura, 1 = Limited visceral pleura involvement (no involvement of mesotelial layer), 2 = Limited visceral pleura involvement (beyond the mesotelial layer)), EGFR: Epidermal Growth Factor Receptor.

The median surveillance time was 60 months (IQR 46-75). During this period, 54 patients experienced disease recurrence, including 26 local and 28 distant relapse, respectively. Pathological analysis indicated that tumor size was larger in patients with recurrence, particularly in those with local recurrence (25.5 mm vs 23.5 mm in patients with distant recurrence or 20 mm in those who remained relapse-free; p=0.028). STAS was more frequently observed in patients with distant recurrence (67.9% vs 42.3% and 46.5% in distant, locoregional, and no recurrence, respectively). A higher proportion of G3 tumors was found in the distant recurrence group (44.4% vs 24% and 20.9%). TILs were less common in patients with local recurrence (38%), while nearly 60% of patients with distant recurrence exhibited more than 10% tumor lymphocytic infiltration (p=0.056). The disease stage distribution was comparable among patients with distant, local or no relapse. The EGFR mutation harbored in 47 cases, accounting for almost the 20% of the sample. In detail, only 6 of those patients had a distant failure (roughly one in five patients with cancer spreading far from the surgical edge) (**Table 3**).

**Table 3:** Cross-table comparing pathological and microscopical data among those who had local or distant relapse and those who remained disease-free.

Characteristic	N	Disease-Free N = 203	Local recurred N = 26	Distant rec. N = 28	p-value
p-stage	256				
I		194 (96%)	24 (92%)	25 (89%)	0.21
IIA		8 (4%)	2 (7.7%)	3 (11%)	
pT size (mm)	257	20.00 (15.00, 27.00)	25.50 (20.00, 30.75)	23.50 (15.00, 30.50)	0.028
STAS	256	94 (46.5%)	11 (42.3%)	19 (67.9%)	0.086
Grading score	253				0.026
G1 and G2		159 (79.1%)	19 (76.0%)	15 (55.6%)	
G3		42 (20.9%)	6 (24.0%)	12 (44.4%)	
Necrosis	254	26 (12.9%)	2 (7.7%)	7 (25.9%)	0.117
TILs	254	125 (62.5%)	10 (38.5%)	18 (64.3%)	0.056
Fibrosis	233	97 (53.0%)	11 (45.8%)	15 (57.7%)	0.698
LVI	255	43 (21.4%)	4 (15.4%)	10 (35.7%)	0.156
Surgical Margin (SM)	229	25.00 (15.00, 40.00)	30.00 (22.50, 40.00)	20.00 (12.50, 29.00)	0.082
Adequate SM	257	135 (66.5%)	20 (76.9%)	18 (64.3%)	0.530
EGFR mutation	257	39 (19.2%)	2 (7.7%)	6 (21.4%)	0.324
Adjuvant treatment	257	5 (2.5%)	3 (11.5%)	3 (10.7%)	0.020

N (%) or median (IQR). Comparisons choose the Kruskal-Wallis rank sum test or the Pearson's Chi-squared test when appropriate.

**Figure 1:**
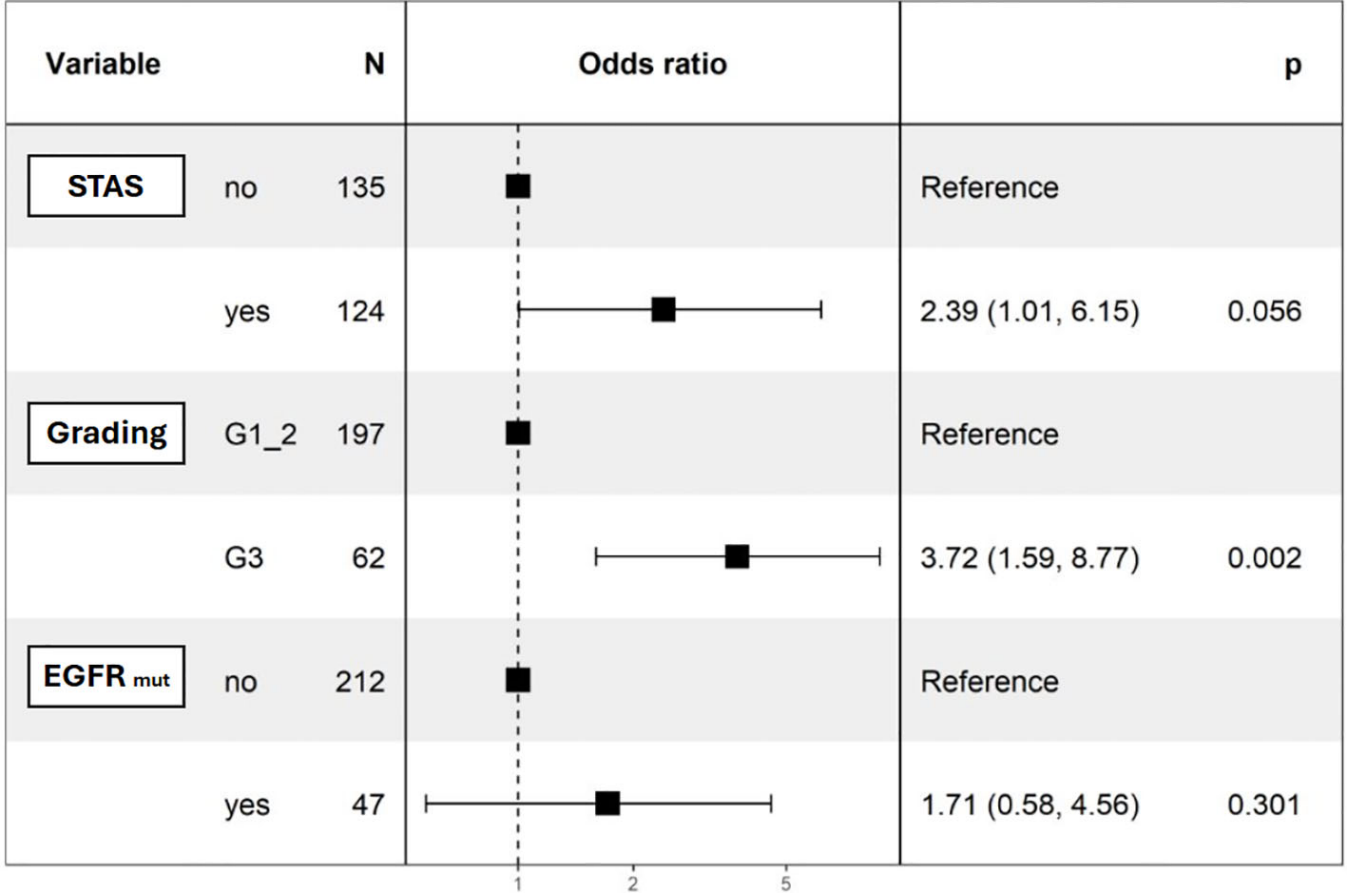
Forest-plot for the EGFR-adjusted model. .

Univariable logistic regression revealed significant associations between distant recurrence and surgical margin (p=0.019), necrosis (p=0.001), G3 grading (p=0.044), and alveolar diffusion (p=0.018). LVI showed a trend toward significance (p=0.054) (**Table 4**). Multivariable logistic regression analysis was divided into two models. The first accounted for pathological predictors only (Parsimonious model - PM) wilst the second also considered the EGFR molecular feature according to the mutational status (EGFR-adjusted Model – EFGR-M). Both of them identified histologic grade as a significant predictor of distant relapse (OR 3.62, 95% CI 1.56-8.38, p<0.01 and OR 3.72, 95% CI 1.59-8.77, p<0.01 respectively). STAS showed a marginal association (OR 2.32, 95% CI 0.95-5.67, p=0.07) in the PM and almost a significance in the EGFR-M (OR 2.39, 95% CI 1.01-6.15, p=0.06). The EGFR status did not influence distant recurrence in our analysis (OR 1.71, 95% CI 0.58-4.56, p=0.301) (**Table 5**; **Figure 1**).

**Table 4:** Univariate logistic regression for distant recurrence.

	Univariate Analysis			
Predictor	Odds-Ratio	Lower CI	Upper CI	p-value
Size	1.02	0.98	1.05	0.353
Surgical Margin	0.96	0.93	0.99	0.019
Grading (G3 vs not G3)	3.87	1.67	8.99	0.001
LVI	2.29	0.96	5.26	0.054
Fibrosis	1.06	0.46	2.53	0.885
TILs	1.16	0.52	2.74	0.720
Tumor necrosis	2.66	0.97	6.67	0.044
STAS	2.85	1.24	7.15	0.018
Adequate margin	0.67	0.30	5.26	0.332
SUVmax (2.5)	1.96	0.72	6.92	0.231

**Table 5:** Multivatiate Logistic Regression Models for Distant Recurrence.

	Parsimonious Multivariate Analysis				EGFR-adjusted Multivariate Analysis			
Predictor	OR	Lower CI	Upper CI	p-value	OR	Lower CI	Upper CI	p-value
Grading (G3 vs not G3)	3.62	1.56	8.38	<0.01	3.72	1.59	8.77	0.002
STAS	2.32	0.95	5.67	0.07	2.39	1.01	6.15	0.056
EGFR mut	–	–	–	–	1.71	0.58	4.56	0.301

On the left side it is reported the model that encompasses only pathological variables (Parsimonious). On the right side the model was implemented accounting for EGFR mutational status (a clinically relevant molecular variable).

**Figure 2:**
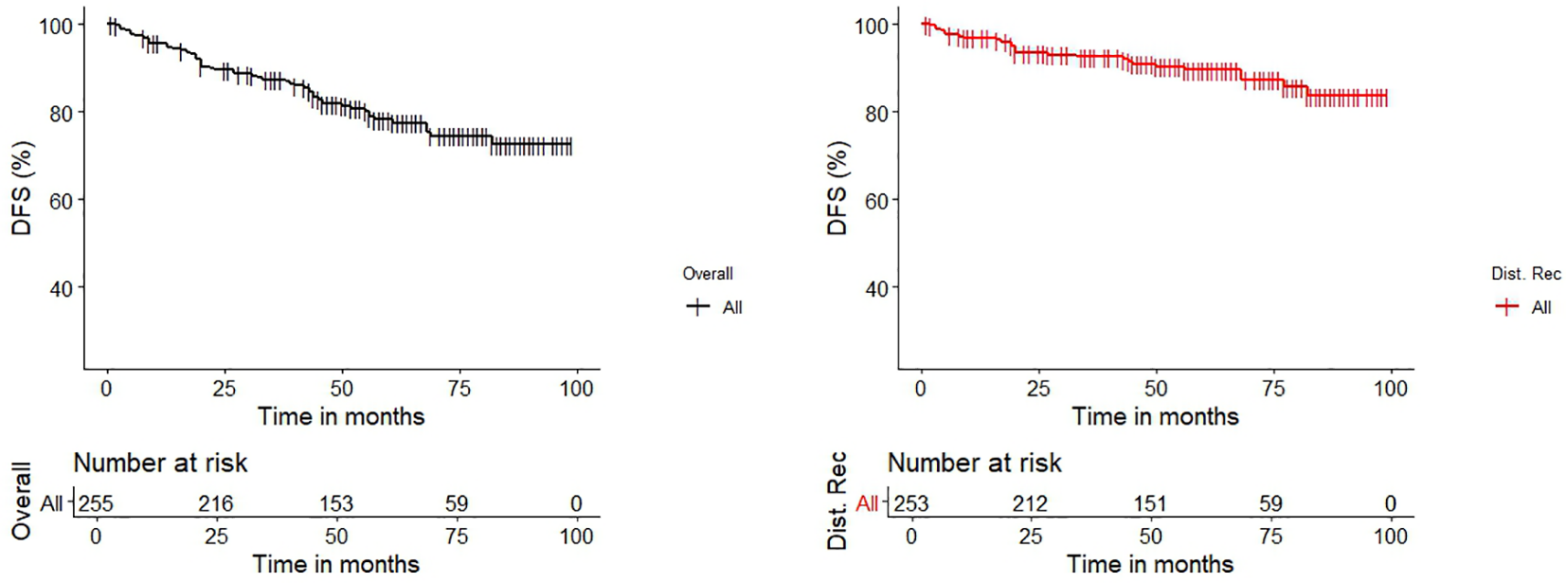
Kaplan-Meier Estimates graphs for Disease-Free Survival (DFS) of the entire cohort (*black line*) and for the cohort of patients which had distant recurrence after curative-intent surgery (*red line*).

In a median surveillance period of 54 months (IQR 40-73), the 96%, 87% and 78% of patients remained overall disease-free at 1-, 3-, and 5-years after surgery. More in detail, taking into account only those who were diagnosed with distant failure during the surveillance, which lasted a median of 20 months (IQR 9-45), they showed a disease-free survival of 97%, 92% and 89% (**Figure 2**).

## 4 Discussion

Despite complete surgical resection with clear margins, postoperative recurrence in stage I-IIA NSCLC remains a major clinical concern. According to cancer biology paradigms, a localized neoplasm should theoretically remain in an equilibrium phase and surgery should be considered a curative treatment (8). However, disease relapse after surgery in early-stage resected NSCLCs is widely reported which suggests that unforeseen microscopic tumor cells may already exist beyond the resection edge at the time of operation. This is why running a scheduled postoperative surveillance is fundamental, particularly within the first two years (9, 10).

In fact, the progressive decline of recurrence-free survival spanning from stage IA to stage III reinforces the correlation between tumor size and relapse rates (5). However, small adenocarcinomas (ADCs) can exhibit aggressive behavior as well, even in early stages, calling for a clearer and refined approach to recurrence risk stratification, going beyond the mere tumor size feature (10–12). While surgical margins have been widely studied, several findings suggest that recurrence risk may also be influenced by microscopic tumor characteristics, regardless of the anatomical resection technique (13–18).

It is plausible that additional, yet unidentified, risk factors of the tumor microenvironment should contribute to ensure the cancer spread beyond the adequate surgical margin. For example, peritumoral structures, such as vessels, lymphatics, and the alveolar tissue, are progressively invaded by growing lung cancer in certain manners that are still only partially understood (19), and this process may play an underestimate role in locoregional or distant failure after a curative-intent surgery (20, 21).

Our findings indicate that STAS, necrosis, histological grade, and surgical margins were significant predictors in the univariable analysis. Notably, our multivariable analysis identified histologic grade as the strongest predictor of distant relapse, with STAS demonstrating only a marginal association in the P-M (p=0.07) and a better fit in the EGFR-M (p=0.05). This further encourages reassessment of the relative importance of these factors in a preoperative setting. These results support previous studies that have identified STAS as a prognostic marker for recurrence (22), but at the same time our findings suggest that alveolar spread may be related to the tumor dedifferentiation (G3). This distinction is critical, as it may influence risk stratification and postoperative management, particularly in patients with STAS-positive, high-grade tumors. Furthermore, tumor necrosis and poorly differentiated histology are associated with increased metabolic and mitotic activity, indicative of aggressive disease with potential for a rapid completion of the epithelial-to-mesenchymal transition (23–25). These features suggest that, irrespective of tumor size and surgical margin, tumor microscopic aggressiveness can be the soil for a distant recurrence even when adequate curative-intent surgery has been performed.

Our results should be interpreted cautiously due to the relatively small sample size and low number of events (only 28 patients with distant recurrence), but similar findings have been reported in the literature. For instance, Fick et al. (26) identified PET-CT uptake, G3 differentiation, LVI, and visceral pleural invasion as predictors of recurrence in stage I ADCs. In another study, Fick et al. (27) found that late recurrence in stage I-III ADCs was associated with pathologic stage, LVI, pleural involvement, and dedifferentiated histology. Furthermore, Wang et al. demonstrated that histologic grade is a better predictor of distant metastasis than tumor stage in early-stage ADCs (28). Our predictors align with Wang’s study, as tumor grade was a more reliable predictor of distant metastasis than tumor size in our analysis.

Finally, LVI has long been considered an independent pathological prognostic factor for recurrence. In this study, LVI showed only a trend toward significance, while in a previous study from our group, LVI was associated with both overall recurrence and survival, with STAS lacking significance (22, 29–33). This discrepancy suggests that analysing recurrence as a single event (made of local and distant recurrence together) may obscure important differences in postoperative tumor spread process, reinforcing the need for stratified risk assessment.

Although molecular profiling was not the primary endpoint of this study, it is worth noting that our center was already performing EGFR mutational testing in early-stage resected lung adenocarcinoma during the study period (2016–2020), anticipating current precision medicine approaches. Among the 54 patients who experienced recurrence, 8 harbored EGFR mutations. While this represents a minority of recurrent cases, had these patients been treated today, they might have been eligible for adjuvant Osimertinib as per the ADAURA trial (34), potentially delaying or preventing recurrence. For this reason, a tailored analysis was performed, showing no influences of EGFR status in the multivariate model for distant recurrence in our study.

Our study aligns with the main literature, highlighting the evolving landscape of postoperative management and the importance of integrating histologic, clinical, and molecular risk factors to guide adjuvant strategies in early-stage disease.

This study has some limitations that should be outlined. As a single-center, retrospective analysis, the findings could be influenced by selection bias and may not be fully generalizable to other institutions or broader populations. Additionally, the relatively small sample size and the limited number of distant failures (only 28 cases) may have reduced the statistical power, potentially affecting the ability to confirm STAS, LVI, and tumor necrosis as independent predictors in multivariable analysis. The lack of data on adjuvant therapies is another potential limitation, as treatment decisions could have influenced recurrence patterns. Despite this, the small contribution of patients receiving adjuvant did not affect our results.

## 5 Conclusions

This study suggests that the G3 histologic grade in resected stage I-IIA ADCs is a key predictor of distant recurrence, calling for a closer postoperative surveillance in these patients. While STAS, LVI, and tumor necrosis were associated with recurrence, their independent prognostic value remains uncertain, likely due to sample size limitations. These findings reinforce the importance of tumor aggressiveness beyond traditional staging parameters and underscore the need for refined risk stratification models to optimize postoperative surveillance.

## Data Availability

The datasets presented in this article are not readily available because Italian privacy policy does not allow to share patients’ records in public. Requests to access the datasets should be directed to Prof. Andrea Dell’Amore, andrea.dellamore@unipd.it.
