# Supplementary material for: Histologic grade and STAS as key predictors of distant recurrence in resected early-stage lung adenocarcinoma: a single-center study
Source: Front Oncol. 2025 Sep 15;15:1626863. doi: 10.3389/fonc.2025.1626863 (PMC12476987; doi:10.3389/fonc.2025.1626863)
Supplement: Supplementary file 1 [file Table1.docx]

# Supplementary Material

**Table S1.** Crosstable comparing perioperative and pathological data among those who recurred and those who remained disease-free.

| ***Demographical and perioperative variables*** |  |  |  |  |
| --- | --- | --- | --- | --- |
| **Characteristic** | N | No recurrence  N = 203 (79%) | Recurrence N = 54 (21%) | p-value |
| **CLINICAL DATA** |  |  |  |  |
| **Age at surgery** | 257 | 69 (62, 75) | 70 (63, 75) | 0.28 |
| **Gender** (Females) | 257 | 107(53%) | 23 (43%) | 0.19 |
| **BMI** | 257 | 26.2 (23.4, 28.8) | 25.1 (22.8, 27.8) | 0.33 |
| **Diabetes** | 257 |  |  | 0.10 |
| No  Yes |  | 182 (90%)  21 (10%) | 44 (81%)  10 (19%) |  |
| **Hypertension** | 257 |  |  | 0.73 |
| No |  | 88 (43%) | 22 (41%) |  |
| Yes |  | 115 (57%) | 32 (59%) |  |
| **Smoking history** | 257 |  |  | 0.92 |
| No |  | 54 (27%) | 14 (26%) |  |
| Yes |  | 149 (73%) | 40 (74%) |  |
| **COPD**  No | 257 | 174 (86%) | 50 (93%) | 0.18 |
| Yes |  | 29 (14%) | 4 (7.4%) |  |
| **FVC %** | 247 | 99 (88, 113) | 102 (92, 107) | 0.96 |
| **FEV1 %** | 247 | 97 (82, 113) | 104 (91, 113) | 0.17 |
| **DLCO/VA %** | 244 | 78 (66, 90) | 77 (68, 87) | 0.73 |
| **Operative time (min)** | 255 | 130 (100, 155) | 120 (90, 150) | 0.23 |
| **Median surveillance time (months)** | 255 | 61 (48, 77) | 55 (44, 70) | 0.13 |
|  |  |  |  |  |

*Abbreviations*: N (%) or median (IQR). Comparisons choose the Kruskal-Wallis rank sum test or the Pearson's Chi-squared test or Wilcoxon’s test when appropriate.

**Table S2.** Crosstable comparing pathological data among those who recurred and those who remained disease-free.

| ***Pathological variables*** |  |  |  |  |
| --- | --- | --- | --- | --- |
| **Characteristic** | N | No recurrence  N = 203 (79%) | Recurrence N = 54 (21%) | p-value |
| **PATHOLOGICAL DATA** |  |  |  |  |
| **p-stage I** | 257 | 194 (96%) | 50 (93%) | 0.48 |
| **Median T SUVmax** | 241 | 4.0 (2.3, 7.1) | 4.9 (3.1, 7.8) | 0.13 |
| **STAS** | 256 | 94 (47%) | 30 (56%) | 0.24 |
| **Surgical Margins (mm)** | 229 | 25 (15, 40) | 25 (15, 34) | 0.55 |
| **Histotype** | 256 |  |  | >0.99 |
| lepidic |  | 21 (10%) | 6 (11%) |  |
| solid |  | 17 (8.4%) | 4 (7.4%) |  |
| others |  | 164 (81%) | 44 (81%) |  |
| **Necrosis** | 254 | 26 (13%) | 9 (17%) | 0.45 |
| **TILs>10%** | 254 | 125 (62%) | 28 (52%) | 0.16 |
| **Fibrosis** | 233 | 97 (53%) | 26 (52%) | 0.90 |
| **LVI** | 255 | 43 (21%) | 14 (26%) | 0.48 |
| **PLI** | 234 |  |  | 0.42 |
| PL0 |  | 77 (41%) | 18 (38%) |  |
| PL1 |  | 97 (52%) | 29 (60%) |  |
| PL2 |  | 12 (6.5%) | 1 (2.1%) |  |
| **N° resected lymphonodes (n)**  N1 lymphonodes  N2 lymphonodes | 255  255  255 | 8 (6, 12)  4 (3, 6)  4 (3, 6) | 8 (7, 12)  4 (2, 5)  5 (4, 7) | 0.90  0.17  0.27 |

*Abbreviations*: N (%) or median (IQR). Comparisons choose the Kruskal-Wallis rank sum test or the Pearson's Chi-squared test or Wilcoxon’s test when appropriate. STAS: Spread Through Air Spaces; TILs: Tumor Infiltrating Lymphocytes; LVI: lympho-vascular invasion; PL: Pleural invasion (0 = Neoplasm-free pleura, 1 = Limited visceral pleura involvement (no involvement of mesothelial layer), 2 = Limited visceral pleura involvement (beyond the mesothelial layer).
